# The medial prefrontal cortex is required for prospective navigation in spatial sequential memory

**DOI:** 10.3389/fnbeh.2026.1930201

**Published:** 2026-09-15

**Authors:** Jihyun Lee, Eunsol Cho, Yong Sang Jo

**Affiliations:** School of Psychology, Korea University, Seoul, Republic of Korea

**Keywords:** excitotoxic lesion, medial prefrontal cortex, prospective navigation, spatial sequential memory, water maze

## Abstract

Goal-directed spatial navigation depends not only on the retrieval of past experiences but also on the prospective representation of future paths. The medial prefrontal cortex (mPFC) is well-established as a critical region for executive functions, particularly in supporting goal-directed behavior and prospective information processing. Although recent electrophysiological recordings show that mPFC neuronal ensembles exhibit predictive codes for future movement directions, a clear behavioral demonstration of whether this region is causally required for prospective navigation remains to be determined. To address this question, we developed a novel spatial sequential memory task using a modified water maze, in which the escape platform moved along a circular path by a constant angle and direction across trials within each day. Using this paradigm, functional dissociation within the prefrontal cortex was investigated by targeting bilateral excitotoxic lesions to two distinct subregions: the mPFC and the anterior cingulate cortex (ACC). Our results demonstrated that mPFC lesions, but not ACC lesions, selectively disrupted the ability to predict and navigate toward the subsequent escape platform location. Furthermore, this behavioral deficit, characterized by disorganized search patterns, occurred without impairment of retrospective navigation to the previously visited location. These findings suggest that the mPFC is essential for prospective spatial navigation, integrating sequential rules with spatial information to generate future trajectories.

## 1 Introduction

The medial prefrontal cortex (mPFC) is widely recognized as a critical neural substrate for mediating executive functions, including working memory, decision-making, behavioral flexibility, and planning (Ragozzino et al., 1999; Miller and Cohen, 2001; Bissonette et al., 2013). While its general involvement in planning is well-documented (Owen et al., 1990; Granon and Poucet, 1995), successful goal-directed navigation in complex environments relies on a more specialized, higher-order capacity. Importantly, this process requires more than merely retrieving spatial memory or temporarily maintaining task-relevant information. Planning in this context depends on a proactive, prospective component of information processing. This function enables an organism to predict the most adaptive responses based on past experiences (Euston et al., 2012), actively organizing and computing future navigational trajectories toward the goal.

Numerous studies have employed *in vivo* electrophysiological recordings during goal-directed navigation tasks (Jung et al., 1998; Pratt and Mizumori, 2001; Hok et al., 2005; Rich and Shapiro, 2009). Recently, these investigations have identified anticipatory activity and prospective coding in mPFC neuronal ensembles, showing that prefrontal networks predict upcoming choices and future trajectories (Burton et al., 2009; Ito et al., 2015; Stout and Griffin, 2020). However, despite extensive reports of prospective neural codes, whether the mPFC is causally required for executing these prospective strategies remains unclear. Furthermore, it is unknown which specific prefrontal subregion mediates this predictive computation. Within the rodent prefrontal cortex, both the mPFC and the anterior cingulate cortex (ACC) serve as critical regions for higher-order cognitive function, yet they play functionally dissociable roles (Bissonette et al., 2013). Therefore, it is necessary to distinguish their causal roles to identify which region mediates the prospective components of navigation.

In the present study, we developed a modified Morris water maze paradigm specifically designed to examine prospective spatial navigation. In this task, the escape platform was moved along a circular path by a fixed angle across consecutive trials, requiring the animals to predict the subsequent location using this sequential logic. With this approach, to determine the causal role of these prefrontal subregions in prospective spatial navigation, neurotoxic lesions were induced in either the mPFC or the ACC. We found that lesions to the mPFC, but not the ACC, significantly impaired the ability to predict the subsequent escape platform locations. Taken together, these findings provide direct behavioral evidence that the mPFC is causally required for prospective information processing, which generates navigational trajectories for goal-directed behavior based on sequential logic.

## 2 Materials and methods

### 2.1 Animals

A total of 57 adult male Sprague Dawley rats (7 weeks old), obtained from Orient Bio (Kyunggi-do, Korea), were utilized for all experiments. The rats were acclimated to the laboratory environment for 1 week prior to surgery. They were housed individually in standard Plexiglas cages within a controlled facility (temperature of 24 °C ± 2 °C and humidity of 50%) under a 12-h light/dark cycle, with food and water available *ad libitum*. Behavioral tasks were conducted during the dark phase of the cycle. All experimental procedures were conducted in accordance with the guidelines of the Korea University Institutional Animal Care and Use Committee.

### 2.2 Surgery

Animals were deeply anesthetized with sodium pentobarbital (60 mg/kg, i.p.) and placed in a stereotaxic apparatus (David Kopf Instruments, Tujunga, CA). The scalp was incised, and the skull was leveled so that bregma and lambda were positioned on the same horizontal plane. Following the drilling of small burr holes, bilateral neurotoxic lesions were induced by infusing 0.5 μl of ibotenic acid per site [5 μg/μl dissolved in phosphate-buffered saline (PBS); Sigma, St. Louis, MO] or an equal volume of PBS alone. Due to the greater anterior–posterior extent of the ACC relative to the mPFC, ibotenic acid was administered at two separate stereotaxic coordinates. The solution was infused at a constant rate of 0.15 μl/min via a 30-gauge stainless steel injection needle connected to an infusion pump (model 101; KD Scientific, Holliston, MA). The toxin was delivered at the following stereotaxic coordinates: (1) mPFC lesions: 3.5 mm anterior, ±0.7 mm lateral, and 3.5 mm ventral to bregma; (2) ACC lesions: (a) 1.8 mm anterior, ±0.6 mm lateral, and 2.2 mm ventral to bregma; (b) 2.2 mm anterior, ±0.6 mm lateral, and 2.4 mm ventral to bregma (Paxinos and Watson, 1998). The injection needle was left in the area 10 min after the injection to allow the solution to diffuse from the injection site. Following surgery, animals were given a recovery period of at least 7 days and were handled daily during this period.

### 2.3 Water maze apparatus

Behavioral testing was performed in a circular metal water maze (157 cm in diameter, 60 cm in height) filled with water maintained at 24 °C ± 1 °C and rendered opaque with black food coloring to hide the platform and increase contrast with the rat. A transparent Plexiglas escape platform (10 cm in diameter, 47 cm in height) was located in one quadrant of the pool and submerged 3 cm below the water level. The maze was located at the center of a well-lit testing room and surrounded by black curtains placed approximately 50 cm from the pool edge. Four distinct visual cues were attached to the curtains and served as distal spatial landmarks. The swimming trajectories of the animals were recorded using an overhead video camera and analyzed with an automated tracking system (SmarTrack; Smartech, Madison, WI).

### 2.4 Behavioral tasks

#### 2.4.1 Spatial sequential memory task

The spatial sequential memory (SSM) task, a modified version of the hidden platform Morris water maze (Morris et al., 1982), was developed to assess prospective spatial navigation. In this task, rats were required to swim and find an escape platform positioned along a circular path with a 47 cm radius from the center of the maze. In each daily session, the platform location for the first trial was determined randomly along this path. For the subsequent trials, the platform was relocated from its initial position according to a fixed directional rule. The platform moved consistently in either a clockwise or counter-clockwise direction (counterbalanced across subjects) with a fixed angular displacement of 27° between trials (Figure 1A). Once assigned, the movement direction remained constant throughout the experiment for each rat. This 27° central angle represents the angular distance between the current and subsequent platform centers, measured from the center of the maze. In a pilot study, when the platform was relocated to an immediately adjacent location in subsequent trials, animals frequently found the next platform accidentally through perseverative searching around the previous platform location. By maintaining a linear gap of approximately 10 cm between the edges of consecutive platforms, a distance corresponding to one platform diameter, this setup demanded intentional sequence-based navigation, requiring the animals to acquire the spatial logic of the task to prospectively navigate to the next platform location. Consequently, this geometric configuration ensured the outer edge of the platform remained at a constant distance of approximately 26.5 cm from the maze wall. The training consisted of four trials per day. At the start of each trial, rats were placed in the water facing the maze wall from a randomized starting position. Each trial ended once the rats found the platform; if the rats failed to find it within 90 s, they were guided to it by an experimenter. Task performance was evaluated by measuring the escape latency, defined as the time required for the rat to find the platform. The rats remained on the platform for 30 s to observe the surrounding distal landmarks and encourage the real-time processing of spatial information. Subsequently, they were transferred to a holding cage for a 3 min intertrial interval (ITI) before starting the next trial from a different position. At the end of each training session, the rats were dried and returned to their home cage.

**FIGURE 1:**
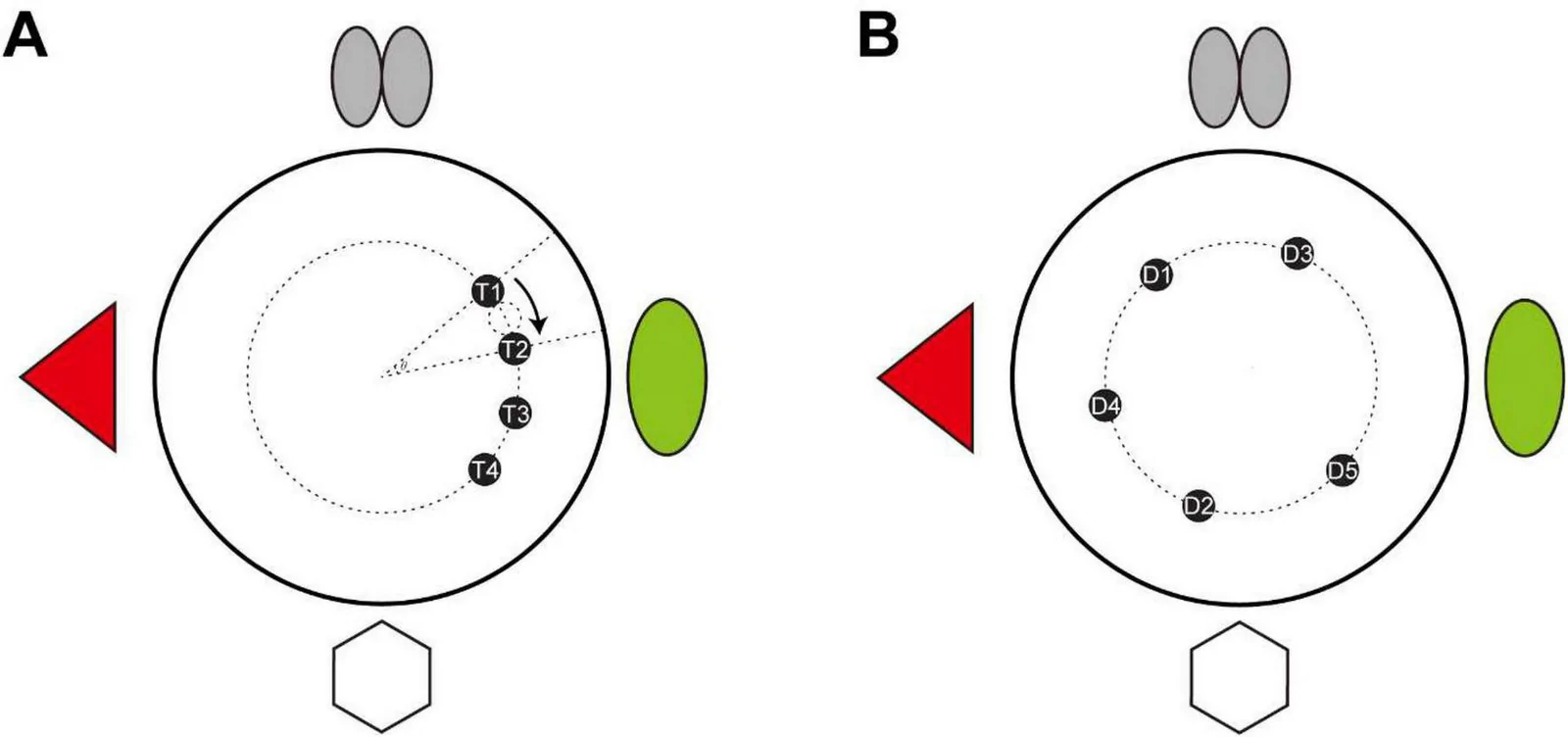
Schematic of the spatial sequential memory (SSM) task paradigm. **(A)** Four distinct visual cues surrounding the pool served as distal spatial landmarks for navigation. The platform was positioned along a circular path (radius: 47 cm) from the center of the maze. Within each day, the platform moved with a fixed angular displacement (θ = 27°) between trials (T1–T4). The arrow indicates the consistent directional movement for subsequent trials. **(B)** To validate the SSM task for the sequential group, the initial platform location was changed daily (D1–D5). For the random group, the same four platform locations were used within each daily session, but the sequence of relocations was pseudorandomized to prevent the acquisition of any spatial sequence.

#### 2.4.2 Spatial working memory task

While the maze and its surrounding setup were identical to those used in the SSM task, the spatial working memory (SWM) task was employed to specifically assess retrospective navigation based on short-term spatial memory (Steele and Morris, 1999). The platform location was altered daily in a pseudorandom manner, ensuring that the same quadrant was not used on two successive days. Within a single day, the platform remained in the same location across all four trials. The rats were required to find the platform location and repeatedly navigate to that same location in the subsequent three trials. Each session consisted of four trials with a 5 min ITI, during which rats were kept in holding cages. Escape latency across trials was measured to assess the rats’ ability to retrospectively navigate to the previously visited location.

### 2.5 Experimental procedures

Prior to the behavioral tasks, all rats underwent a 7-days habituation period to acclimate to the water maze environment without an escape platform. During this period, each rat underwent three trials per day with a 1-h ITI, swimming freely for 90 s in each trial. This prolonged 7-days habituation was designed to reduce any task-related anxiety and allow the animals to fully acquire the spatial context by exposing them to the maze and its distal landmarks.

For all behavioral tasks, each trial began by placing the rat in the water facing the maze wall. The starting position was randomized among three of the four quadrants (north, east, south, and west), excluding the quadrant containing the platform, to ensure that the animals relied on spatial learning rather than a fixed swimming pattern.

In the first experiment, the validity of the SSM task was evaluated by comparing the performance of sequential (*n* = 11) and random (*n* = 11) groups. The sequential group was trained for 5 days according to the constant directional rule as described above. In contrast, the random group was exposed to the identical four platform locations within each daily session, but the sequence of relocations was pseudorandomized to prevent the acquisition of any spatial logic. For both groups, a brief ITI was maintained between trials to allow for the relocation of the platform to its next position.

In the second experiment, the differential involvement of the mPFC and the ACC in the predictive component of the SSM was investigated. Bilateral excitotoxic lesions were induced in either the mPFC (*n* = 11) or the ACC (*n* = 11) via ibotenic acid infusion. A sham-operated group (*n* = 13) served as a control, receiving bilateral infusions of PBS into either the mPFC (*n* = 7) or the ACC (*n* = 6). Following a one-week recovery period, the SSM was conducted for 5 days using the same protocol as Experiment 1 to determine the functional requirement of each subregion in prospective spatial navigation.

In the third experiment, the SWM task was utilized to examine whether lesions to these prefrontal subregions selectively impaired prospective navigation or induced a broader deficit in spatial working memory. The same groups used in the second experiment (Sham, mPFC lesion, and ACC lesion) were assessed for 3 days. Unlike the SSM task, the SWM task required daily updates of the platform location, following the protocol described above to assess spatial working memory performance.

### 2.6 Histology

After the behavioral experiments were completed, histological analyses were conducted to verify lesion sites. All rats were deeply anesthetized with an overdose of sodium pentobarbital (80 mg/kg) and transcardially perfused with PBS followed by 4% paraformaldehyde (PFA) in PBS (pH 7.4). The brains were removed from the skull, postfixed overnight at 4 °C in the same fixative, and then transferred to 30% sucrose in PBS at 4 °C until they sank. Brains were sectioned coronally at 50 μm using a freezing microtome (SM2000R; Leica). The sections included the mPFC and ACC. Sections were mounted onto gelatin-coated slides, stained with cresyl violet, coverslipped with Permount, and examined using light microscopy.

To quantify the extent of the lesion, four coronal sections for the mPFC and five for the ACC were selected at approximately equal intervals, covering the rostrocaudal extent of each target region (from +4.20 to +3.00 mm and from +2.52 to +1.56 mm anterior to bregma, respectively). Lesion extent was quantified using ImageJ by measuring the area of neuronal loss and the total area of the mPFC or ACC in each section. The percentage of damage was calculated as the ratio of lesioned area to total regional area for each section.

### 2.7 Statistical analysis

For all experiments, escape latency was initially analyzed using a three-way mixed-design analysis of variance (ANOVA), which included within-subjects factors (day and trial) and a between-subjects factor (group). Average swimming speed and entries to the previous platform location were analyzed using a two-way mixed-design ANOVA, which included a within-subjects factor (day) and a between-subjects factor (group). When a significant interaction involving the group factor (e.g., group × trial or group × day) was observed, subsequent simple-effect analyses for each training day (two-way repeated-measures ANOVAs) were justified to further investigate how group differences emerged across training days. When significant interactions or simple main effects were observed, *post hoc* pairwise comparisons were performed. For multiple comparisons among three or more groups, Tukey’s *post-hoc* test was applied. All statistical analyses were conducted using SPSS software (version 29.0; IBM SPSS, Armonk, NY), and *p*-values < 0.05 were considered statistically significant. Unless otherwise stated, all data were presented as mean ± SEM.

## 3 Results

### 3.1 The SSM task effectively validates the assessment of predictive navigation

To confirm that the newly developed SSM task reliably measures spatial sequential memory, we compared the performance of the sequential and random groups in Experiment 1. Prior to the behavioral task, all animals underwent a 7-days habituation period to ensure acclimation to the water maze environment and minimize task-related anxiety, thereby stabilizing behavioral baselines. In the sequential group, while the initial platform location for the first trial of each day was randomly selected (Figure 1B), the platform followed a consistent spatial sequence in subsequent trials. In contrast, the random group was exposed to the identical four platform locations within each daily session, but the sequence of relocations was pseudorandomized, preventing the acquisition of any spatial sequence.

On the first day of training, both groups exhibited a similar within-day reduction in escape latency, suggesting that the sequential group had not yet acquired the transition logic of the platform’s sequential movement (Figure 2A). However, a significant difference in performance emerged from day 2 (*p* < 0.01). The sequential group, after identifying the platform location in the first trial, exhibited a sharp decrease in escape latencies in subsequent trials. In contrast, the random group showed less pronounced improvement, maintaining consistently longer latencies. This significant performance gap between the groups persisted through days 3–5 (*p* < 0.001), demonstrating that the sequential group successfully acquired the platform sequence. Importantly, because the initial platform location on trial 1 was randomly assigned each day, it served primarily as a relearning trial rather than a measure of prospective navigation. To provide a more direct measure of predictive navigation, we conducted a supplementary summary analysis focusing on the average escape latency of trials 2–4. This analysis confirmed that the random group exhibited significantly longer escape latencies than the sequential group (*p* < 0.001), providing evidence for the successful utilization of sequence-based navigation.

**FIGURE 2:**
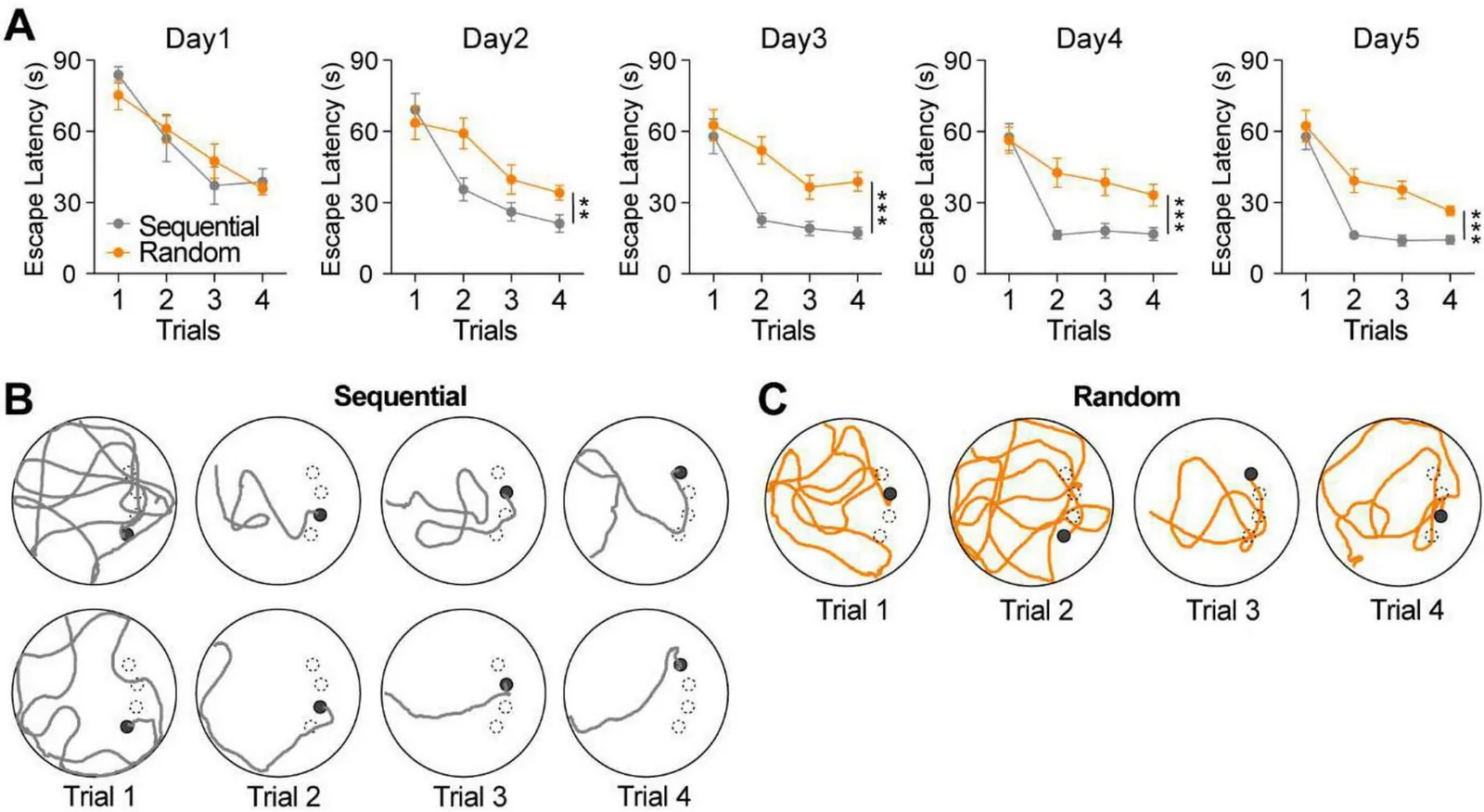
Behavioral validation and trajectory analysis of the SSM task. **(A)** Escape latency during 5 days of training (*n* = 11 per group). The Sequential group showed a significantly more rapid reduction in escape latency compared to the Random group. A three-way mixed-design ANOVA revealed significant Trial × Group [*F*(3, 60) = 7.80, *p* < 0.001] and Day × Group [*F*(4, 80) = 3.39, *p* = 0.013] interactions, justifying subsequent simple-effect analyses for each training day. Two-way repeated measures ANOVAs revealed a significant main effect of trial across Days 1–5 [Day 1: *F*(3, 60) = 20.80, *p* < 0.001; Day 2: *F*(3, 60) = 20.04, *p* < 0.001; Day 3: *F*(3, 60) = 18.28, *p* < 0.001; Day 4: *F*(3, 60) = 22.19, *p* < 0.001; Day 5: *F*(3, 60) = 47.09, *p* < 0.001]. A significant main effect of group was found on Days 2–5 [Day 2: *F*(1, 20) = 8.41, *p* = 0.009; Day 3: *F*(1, 20) = 31.82, *p* < 0.001; Day 4: *F*(1, 20) = 17.60, *p* < 0.001; Day 5: *F*(1, 20) = 21.19, *p* < 0.001], but not on Day 1 [*F*(1, 20) = 0.02, *p* = 0.892]. Specifically on Day 4, a significant trial × group interaction was observed [*F*(3, 60) = 3.57, *p* = 0.019; *post-hoc* tests at each trial, *p* < 0.01 except for trial 1]. Note that a supplementary summary analysis focusing on trials 2–4 (excluding the unpredictable trial 1) further confirmed a significant main effect of group [*F*(1, 20) = 47.25, *p* < 0.001]. **(B)** Representative swimming trajectories of the sequential group showing two strategies: visiting the previous location (top) and navigating directly to the next predicted location in trials 3 and 4 (bottom). **(C)** Representative swimming trajectories of the random group showing disorganized search patterns. Data are presented as mean ± SEM. **p < 0.01, ***p < 0.001.

The analysis of swimming trajectories from day 5 further elucidated the strategic divergence between the two groups (Figures 2B,C). The sequential group successfully reached the current platform by either visiting its previous location first (Figure 2B top) or navigating directly to the subsequent location based on platform sequence (Figure 2B bottom; especially in trials 3 and 4). In contrast, the random group exhibited disorganized search patterns. Despite visiting the previous location, they lacked the ability to anticipate the subsequent platform position (Figure 2C). These findings confirm the behavioral validity of the SSM paradigm in assessing spatial sequential memory, which measures prospective navigation based on the platform sequence as distinct from simple spatial memory.

### 3.2 The mPFC is required for spatial sequential memory

In Experiment 2, to identify which prefrontal subregion is responsible for the prospective spatial navigation based on sequential logic, we performed selective ibotenic acid lesions in either the mPFC or the ACC. As an excitotoxic agent, ibotenic acid primarily targets neuronal cell bodies while sparing axons of passage (Jo and Lee, 2010). Histological evaluation confirmed the extent of damage induced by these lesions across a series of brain sections. Lesions were defined by regions showing clear neuronal loss and disruption of normal cortical architecture, including attenuation or loss of laminar organization (Figure 3A). The lesion extent remained consistent across subjects, confirming the anatomical specificity of these lesions (Figure 3B). For the mPFC, lesions were primarily centered on the prelimbic (PL) cortex, and the percentage of damage was calculated relative to the combined total area of the PL and infralimbic (IL) cortices. For behavioral analysis, we applied an inclusion criterion requiring a minimum of 70% damage to the target structure, and subjects showing significant excitotoxic spread into adjacent cortical areas were excluded. Quantitative analysis of the included subjects confirmed that the lesions encompassed 77.42% ± 7.81% of the mPFC (ranging from 70.69% to 92.59%) and 76.36% ± 6.76% of the ACC (ranging from 71.53% to 86.32%) (mean ± SD).

**FIGURE 3:**
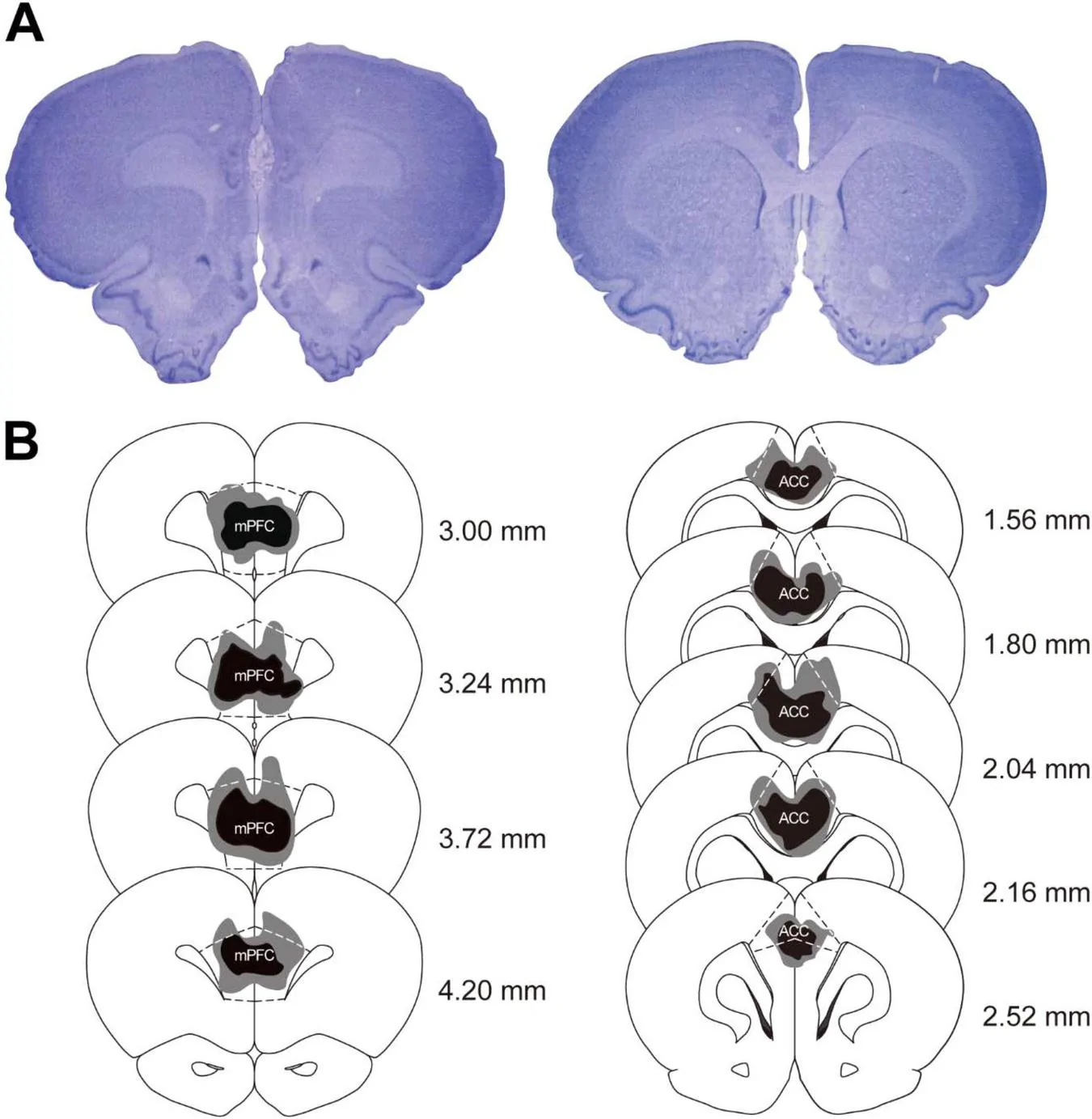
Histological verification of ibotenic acid-induced lesions in the mPFC and ACC. **(A)** Representative photomicrographs of Nissl-stained brain sections illustrate the extent of mPFC and ACC damage following ibotenic acid lesions. **(B)** Schematic reconstructions of the lesion sites indicate the maximum and minimum extents of the lesions with gray and black areas, respectively. The anteroposterior stereotaxic coordinates are shown by the numbers beside the sections (Paxinos and Watson, 1998).

To assess spatial sequential memory, animals with verified mPFC or ACC lesions, along with sham-operated controls, were trained on the SSM task for five consecutive days. As preliminary statistical analyses revealed no significant differences in behavioral performance between the mPFC-sham and ACC-sham subgroups, their data were combined into a single sham group (*n* = 13) for all analyses. Each day, the initial platform location for the first trial was randomly assigned (Figure 4A). In subsequent trials, the platform followed a consistent spatial sequence, moving in either a clockwise or counterclockwise direction relative to its previous location to prompt sequential navigation. To evaluate the overall performance in the SSM task, we initially performed a three-way mixed-design ANOVA on escape latency. While the three-way interaction was not significant, we observed a significant Trial × Group interaction (*p* = 0.009), justifying subsequent simple main effects analyses for each training day. While all groups showed a general trial-dependent reduction in escape latency, significant performance differences emerged between the groups starting from day 2 (Figure 4B). On day 2 and day 3, the mPFC group exhibited significantly longer latencies compared to the sham group (*p* < 0.05). By days 4 and 5, this deficit became more severe, with the mPFC group showing significantly impaired performance compared to both the sham and ACC groups (*p* < 0.05). In contrast, *post hoc* comparisons revealed no significant differences in escape latencies between the sham and ACC groups across all training days (*p* > 0.05).

**FIGURE 4:**
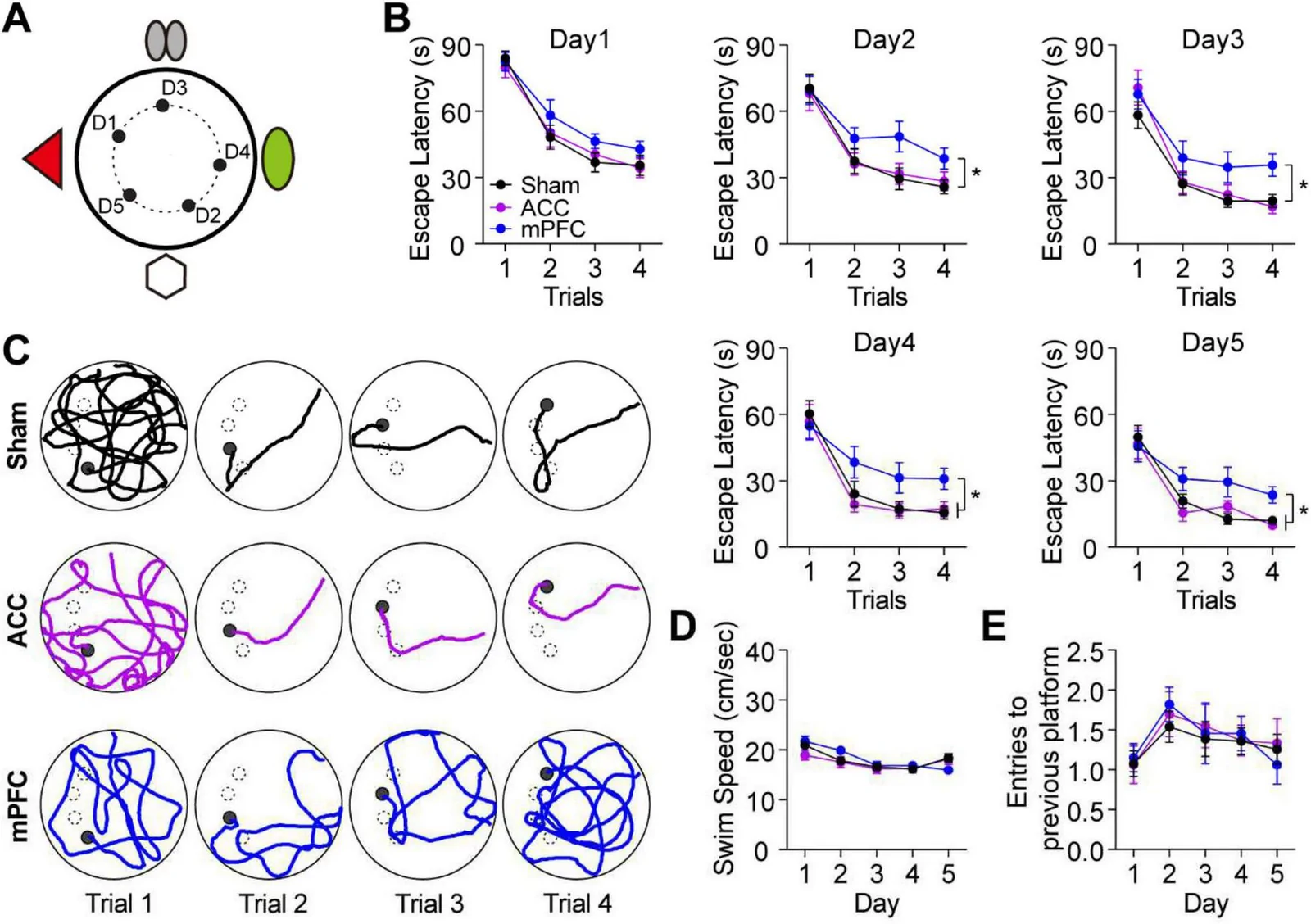
Role of the mPFC and ACC in spatial sequential navigation. **(A)** Schematic of the SSM task, in which the initial platform location was changed daily. **(B)** Escape latency during 5 days of training (Sham, *n* = 13; mPFC and ACC, *n* = 11 each). A three-way mixed-design ANOVA revealed a significant Trial × Group interaction [*F*(6, 96) = 3.04, *p* = 0.009], whereas other interactions involving the group factor were not significant (*p* > 0.05), justifying subsequent simple-effect analyses for each training day. Daily two-way repeated-measures ANOVAs demonstrated a significant main effect of trial across Days 1–5 [Day 1: *F*(3, 96) = 52.81, *p* < 0.001; Day 2: *F*(3, 96) = 29.14, *p* < 0.001; Day 3: *F*(3, 96) = 41.36, *p* < 0.001; Day 4: *F*(3, 96) = 31.47, *p* < 0.001; Day 5: *F*(3, 96) = 32.68, *p* < 0.001] and a significant main effect of group on Days 2–5 [Day 2: *F*(2, 32) = 4.24, *p* = 0.023; Day 3: *F*(2, 32) = 5.02, *p* = 0.013; Day 4: *F*(2, 32) = 5.31, *p* = 0.010; Day 5: *F*(2, 32) = 4.42, *p* = 0.020]. Specifically, the mPFC group showed significantly longer latencies than the Sham group on Days 2 and 3, and also than both the Sham and ACC groups on Days 4 and 5 (*post-hoc* tests, *p* < 0.05). Note that a supplementary summary analysis focusing on trials 2–4 (excluding the unpredictable trial 1) further confirmed a significant main effect of group [*F*(2, 32) = 34.35, *p* < 0.001], with the mPFC group showing significantly longer latencies than both the Sham and ACC groups (*post-hoc* tests, *p* < 0.001). **(C)** Representative swimming trajectories. The Sham (top) and ACC (middle) groups navigated toward the predicted platform location, whereas the mPFC group (bottom) showed disorganized search patterns and a failure to utilize sequential information. **(D)** Swimming speed. There were no significant differences in average swimming speed among the groups across all training days [main effect of group: *F*(2, 32) = 0.38, *p* = 0.689]. **(E)** Entries to the previous platform location. No significant differences were observed among the groups across all training days [main effect of group: *F*(2, 32) = 0.13, *p* = 0.880]. Data are presented as mean ± SEM. *p < 0.05.

Consistent with the analysis of Experiment 1 (Figure 2A), we conducted a supplementary summary analysis focusing on the average escape latency of trials 2–4 to disentangle sequence-based prospective navigation from the relearning effect of trial 1. This analysis confirmed that the mPFC group exhibited significantly longer escape latencies compared to both the sham and ACC groups (*p* < 0.001), underscoring the selective requirement of the mPFC in sequence-based prospective navigation. To determine whether these differences in escape latency were confounded by nonspecific locomotor or motivational alterations, we analyzed the average swimming speed across all training days (Figure 4D). There were no significant differences in overall swimming speed among the groups. This comparable performance indicates that the behavioral deficit in the mPFC group was not due to deficits in locomotor function or motivation.

These findings are further supported by the representative swimming trajectories from day 5. The sham and ACC groups exhibited sequence-based navigation toward the subsequent platform (Figure 4C top and middle), whereas the mPFC group displayed disorganized search patterns (Figure 4C bottom). Indeed, the mPFC group tended to swim toward the previous platform location rather than navigating directly to the subsequent location. Importantly, no significant differences were observed between groups in the mean entries to the previous platform location (Figure 4E). This indicates that the mPFC deficit was not due to an inability to retain the previous platform location, but rather a failure to integrate sequential information for navigation toward the subsequent target. These results demonstrate that the mPFC, but not the ACC, is required for the processing of spatial sequences necessary for prospective navigation.

### 3.3 Spatial working memory is preserved following mPFC or ACC lesions

Next, we tested performance on the SWM task in Experiment 3 to confirm whether mPFC or ACC lesions affected general ability to acquire and retain a new platform location each day. In this task, the platform location was changed daily but remained fixed across the four trials within each day (Figure 5A). All groups showed a reduction in escape latency across trials, with no significant differences observed among the groups throughout the testing period (Figure 5B). These results indicate that mPFC or ACC lesions do not impair the fundamental capacity to acquire and temporarily maintain spatial information, suggesting that spatial working memory is preserved.

**FIGURE 5:**
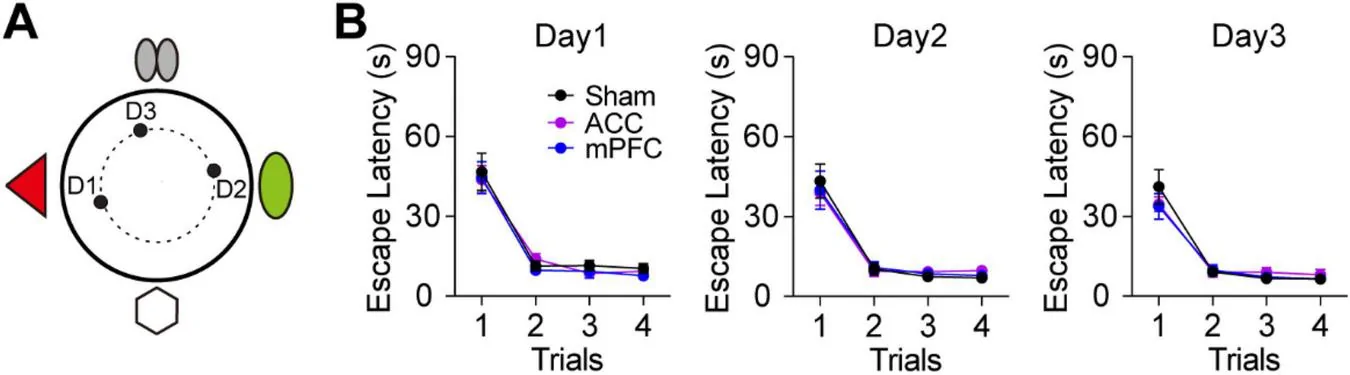
Spatial working memory performance following mPFC or ACC lesions. **(A)** Schematic of the spatial working memory (SWM) task, in which the initial platform location was changed daily. **(B)** Escape latency during 3 days of training (Sham, *n* = 13; mPFC and ACC, *n* = 11 each). All groups showed a reduction in escape latency across trials, with no significant differences observed among the groups. A three-way mixed-design ANOVA revealed a significant main effect of trial [*F*(3, 96) = 149.91, *p* < 0.001], whereas the main effect of group and all interactions involving the group factor were not significant (*p* > 0.05). Data are presented as mean ± SEM.

## 4 Discussion

While correlational observations indicate that mPFC neuronal ensembles exhibit prospective coding for future trajectories (Ito et al., 2015; Stout and Griffin, 2020), its causal requirement for prospective navigation has remained elusive. Using a modified Morris water maze paradigm with an escape platform that moved along a circular path by a fixed angle, we developed the SSM task to examine prospective spatial navigation. We first validated the task by confirming that animals could utilize a consistent spatial sequence to predict subsequent escape platform locations, whereas random platform movement severely disrupted this prospective navigation, resulting in erratic search patterns. Following the behavioral validation, in a subsequent experiment, we induced neurotoxic lesions in either the mPFC or the ACC to determine how different prefrontal subregions mediate this prospective navigation. The ability to predict subsequent platform locations was selectively impaired by mPFC lesions, resulting in disorganized search patterns and longer escape latencies, whereas ACC lesions had no effect. Importantly, this predictive navigation impairment was observed despite intact entries to the previous platform location. These findings demonstrate that the mPFC is required for prospective spatial navigation based on sequential logic.

Previous *in vivo* electrophysiological studies have provided profound insights into how the mPFC encodes goal-directed behavior. Single-unit recordings have revealed that mPFC neurons exhibit anticipatory activity for expected rewards and selectively encode specific goal locations (Pratt and Mizumori, 2001; Hok et al., 2005; Burton et al., 2009). Importantly, the mPFC not only anticipates these specific locations but also prospectively encodes the ongoing spatial trajectories leading to them. Subsequent studies employing discrete choice tasks, such as the T-maze or Y-maze, have demonstrated that mPFC neuronal ensembles exhibit prospective coding through trajectory-dependent firing rate changes that predict an animal’s upcoming left or right turn prior to reaching a decision point (Bai et al., 2012; Ito et al., 2015; Stout and Griffin, 2020). Furthermore, computational population decoding analyses have confirmed that mPFC ensemble activity can predict an animal’s prospective trajectory choices and future actions well before the actual behavioral execution (Ito et al., 2015; Stout and Griffin, 2020; Jeong and Choi, 2026). However, because these electrophysiological and computational approaches are intrinsically correlational, the causal necessity of these predictive signals has remained largely unexplored. In this regard, our modified water maze paradigm provides a distinct methodological advantage. Consistent with these predictive neural representations, by utilizing an excitotoxic lesion approach within our SSM task, we found that mPFC lesions profoundly disrupted the prospective processing required to navigate toward subsequent goals along a circular path. These findings provide direct behavioral evidence that the mPFC is causally required to integrate sequential information to generate future behavioral trajectories during prospective spatial navigation. Thus, we confirmed a specific role for the mPFC in goal-directed behavior (Hok et al., 2005; Euston et al., 2012).

In our study, we observed a functional and anatomical dissociation between the mPFC and the ACC regarding prospective spatial navigation. While mPFC lesions profoundly disrupted the ability to predict platform locations, ACC lesions produced no significant behavioral deficits. This dissociation aligns with evidence that mPFC neurons encode spatial goals, whereas ACC neurons rarely exhibit such firing properties (Hok et al., 2005). Anatomically, the hippocampus, which is crucial for spatial cognitive mapping (O’keefe and Nadel, 1978; Pearce et al., 1998), sends significantly denser direct projections to the ventral mPFC, specifically the PL and IL cortices, compared to the ACC, which receives relatively sparse to moderate hippocampal efferents (Jay and Witter, 1991; Hoover and Vertes, 2007). Therefore, this heavy hippocampal projection provides the critical anatomical basis that enables the mPFC to effectively integrate spatial information and compute future trajectories (Ito et al., 2015; Spellman et al., 2015; Ito, 2018), making it essential for spatial sequential memory. In contrast, the ACC receives relatively sparse spatial input from the hippocampus and is not required for spatial sequential memory. It is highly likely that these two prefrontal subregions contribute to distinct aspects of future-oriented cognition. Specifically, while the mPFC is engaged in replaying spatial representations to predict future trajectories (Kaefer et al., 2020), the ACC primarily mediates general performance monitoring, such as detecting behavioral errors and evaluating recent outcomes to adaptively adjust subsequent actions (Narayanan and Laubach, 2008; Totah et al., 2009; Bryden et al., 2011; Bissonette et al., 2013). Consistent with these distinct but complementary roles, our findings demonstrate that the mPFC is selectively essential for generating future navigational trajectories based on sequential logic.

Importantly, the impairment in predictive navigation occurred despite intact spatial working memory. It is well established that mPFC damage profoundly disrupts spatial working memory across various spatial tasks (Granon et al., 1994; Yoon et al., 2008; Churchwell and Kesner, 2011). However, our previous study demonstrated that extensive prior exposure to the maze environment can preserve this ability despite mPFC dysfunction (Jo et al., 2007). With this in mind, the animals in our study underwent a prolonged 7-days habituation period to fully acquire the spatial context and stabilize behavioral baselines. Under these conditions, the retrospective maintenance of short-term spatial information remained preserved. Indeed, the mPFC group exhibited a comparable number of entries to the previous platform location during the SSM task (Figure 4E) and successfully navigated to the daily assigned platform location during the SWM task (Figure 5B), confirming intact spatial working memory. Therefore, the navigational deficit observed in the current study does not result from an impairment in retrospective memory, but rather reflects a failure in prospective coding, which refers to the ability to further process retained information to compute future navigational actions (Jeong and Choi, 2026). This prospective computation is likely mediated by the active replay of extended spatial trajectories (Kaefer et al., 2020), reflecting the broader role of the mPFC in exerting top-down control over memory representations to guide goal-directed behaviors (Yadav et al., 2024).

Successful performance in the SSM task inherently requires both the acquisition of the sequential rule and its prospective application to navigation. However, the behavioral paradigm used in our study cannot easily dissociate the initial acquisition of the sequential logic from its ongoing prospective navigation. Therefore, while our findings clearly demonstrate that the mPFC is essential for spatial sequential navigation, whether its primary contribution lies in encoding the sequential rule itself or in retrieving and utilizing the learned sequential logic during prospective navigation remains an open question. Further studies utilizing stage-specific neural manipulations during distinct learning phases will be necessary to fully distinguish these possibilities.

In summary, our study demonstrates that the mPFC, but not the ACC, is essential for prospective spatial navigation. While the short-term maintenance of the previously visited locations is preserved independently of the mPFC, generating future navigational trajectories toward a subsequent goal based on sequential logic specifically requires mPFC function. Ultimately, these findings provide evidence for the mPFC as an executive system, confirming that its prospective function represents a fundamental cognitive computation for goal-directed spatial navigation.

## Data Availability

The original contributions presented in this study are included in this article/supplementary material, further inquiries can be directed to the corresponding author.
